# Availability of genotypic and phenotypic drug susceptibility testing for *Mycobacterium tuberculosis* in WHO European Region

**DOI:** 10.5588/ijtldopen.25.0600

**Published:** 2026-10-12

**Authors:** C.U. Köser, D.K. Paspaliari, A.M. Cabibbe, D.M. Cirillo, A. Yedilbayev, S. Ehsani

**Affiliations:** 1 Department of Genetics, University of Cambridge, Cambridge, UK;; 2 European Laboratory Initiative on TB, HIV and Viral Hepatitis, WHO Regional Office for Europe, Copenhagen, Denmark;; 3 Joint Infectious Diseases Unit, Division of Communicable Diseases, Environment and Health, WHO Regional Office for Europe, Copenhagen, Denmark;; 4 Emerging Bacterial Pathogens Unit, IRCCS San Raffaele Scientific Institute, Milan, Italy;; 5 Pandemic Threats, Communicable Diseases and Antimicrobial Resistance Unit, Health Security Division, WHO Regional Office for Europe, Copenhagen, Denmark.

**Keywords:** tuberculosis, *Mycobacterium tuberculosis*, drug resistance, DST, genotypic and phenotypic drug susceptibility testing

## Abstract

**BACKGROUND:**

The WHO European Region has the highest rates of drug-resistant TB globally. Therefore, we studied the availability of genotypic and phenotypic drug susceptibility testing (DST), including targeted next-generation sequencing and whole genome sequencing, for first- and second-line drugs.

**METHOD:**

In 2024, the results of an online survey completed by national and supranational reference laboratories (or equivalent) of 53 countries, territories, and areas of the WHO European Region were reviewed. The availability of DST was analysed using three denominators: 1) the number of countries, territories, and areas for all drugs; 2) the estimated 2024 TB incidence for first-line drugs, including fluoroquinolones; and 3) the estimated 2024 rifampicin-resistant TB incidence for the remaining drugs.

**RESULTS:**

The most important gap in the availability of DST was for pretomanid, the most recently approved anti-TB drug: phenotypic DST was only available for 4% of the estimated incident cases of rifampicin-resistant TB in countries where pretomanid is approved or used programmatically. This contrasted with an availability of phenotypic DST of >98% for bedaquiline, delamanid, and linezolid.

**CONCLUSION:**

The delay between the introduction of novel drugs and the availability of robust DST services should be minimised to avoid intrinsic and selected resistance to novel drugs going unnoticed.

Drug-resistant TB remains a persistent global health concern. Several 6- to 9-month all-oral regimens for rifampicin-resistant TB (RR-TB) are being scaled up, which are expected to improve treatment success while reducing side-effects and costs compared with traditional longer regimens with injectable drugs.^1^ In particular, BPaL(M), the 6-month regimen consisting of bedaquiline, pretomanid, and linezolid with or without moxifloxacin, is projected to be used for most adult patients with RR-TB.^2^ The use of these novel regimens must be accompanied with high-quality drug susceptibility testing (DST) to select the optimal regimen.^3^ This is crucial as resistance to bedaquiline, delamanid, and pretomanid has arisen repeatedly in different genetic backgrounds of the *Mycobacterium tuberculosis* complex prior to the clinical use of these novel agents.^4–7^ Moreover, at approximately 2%, the rate of acquired bedaquiline resistance, the core drug of all WHO-recommended all-oral RR-TB regimens, is an order of magnitude higher than the rate of acquired rifampicin resistance during treatment of drug-susceptible TB, underlining the importance of bedaquiline DST during treatment.^5,7–10^

We conducted a detailed survey of the availability of genotypic DST (gDST), including targeted next-generation sequencing (tNGS) and whole genome sequencing (WGS), as well as phenotypic DST (pDST) in the WHO European Region, given that earlier studies were either outdated or had a more limited scope.^11–15^ A comprehensive overview of the DST availability in this Region is particularly important as it includes some of the countries, territories, and areas with the highest rates of RR-TB among new and retreatment cases worldwide and approximately 55,000 individuals who are estimated to have RR-TB in 2024, which corresponds to 14% of the estimated global incidence.^16^

## METHODS

We sent an online survey to the national and supranational reference laboratories (or equivalent) of 54 of the 56 countries, territories, and areas (53 Member States, Greenland, Kosovo [All references to Kosovo in this document should be understood to be in the context of the United Nations Security Council resolution 1244 (1999).], and Liechtenstein) in the WHO European Region. Liechtenstein, a European Union/European Economic Area (EU/EEA) country, was not covered as it is not a WHO member state. Monaco was excluded as no contact at the relevant laboratory could be identified. Responses from 53 countries, territories, and areas, including all 18 high-priority countries (HPCs) and 29 EU/EEA countries, of which five are also HPCs (Table 1), were received between late February and June 2024, followed by clarifications with some respondents where necessary. No response was received from San Marino. Respondents were asked whether pDST, rapid, commercial gDST assays, and/or WGS were available for patient care or being validated/verified for patient care in their country, territory, or area (i.e., use for research only did not qualify).^17^

**Table 1.** Overview of 53 countries, territories, and areas of the WHO European Region that responded to survey.

HPCs (18)	EU/EEA countries, excluding HPCs (24)	Other countries, territories, or areas (11)
Armenia	Austria	Albania
Azerbaijan	Belgium	Andorra
Belarus	Croatia	Bosnia and Herzegovina
Bulgaria**^A^**	Cyprus	Israel
Estonia**^A^**	Czechia	Montenegro
Georgia	Denmark	North Macedonia
Kazakhstan	Finland	Serbia
Kyrgyzstan	France	Switzerland
Latvia**^A^**	Germany	United Kingdom of Great Britain and Northern Ireland
Lithuania**^A^**	Greece	Greenland
Republic of Moldova	Hungary	Kosovo
Romania**^A^**	Iceland	
Russian Federation	Ireland	
Tajikistan	Italy	
Türkiye	Luxembourg	
Turkmenistan	Malta	
Ukraine	Kingdom of the Netherlands	
Uzbekistan	Norway	
	Poland	
	Portugal	
	Slovakia	
	Slovenia	
	Spain	
	Sweden	

EU/EEA = European Union/European Economic Area; HPCs = high-priority countries.

A EU/EEA members included in the EU/EEA subset of 29 countries.

The following 16 drugs were included for pDST and WGS: first-line (rifampicin, isoniazid, ethambutol, pyrazinamide, and moxifloxacin) and second-line (levofloxacin, bedaquiline, linezolid, clofazimine, cycloserine, delamanid, amikacin, streptomycin, ethionamide/prothionamide, *para*-aminosalicylic acid, and pretomanid), where rifampicin serves as the surrogate for rifapentine and cycloserine as the surrogate for terizidone.^1^ We did not investigate the ability to stratify the level of isoniazid or moxifloxacin resistance.^18^ A combined analysis of the availability of pDST for fluoroquinolones as a class was also carried out. Carbapenems could not be included as no critical concentrations for pDST exist and the genetic basis for resistance is also poorly understood.^19^ Rifabutin was not considered as it does not feature in WHO treatment recommendations.^1^ Information about the testing method and critical concentrations was only gathered for pretomanid pDST given that the WHO had not yet set critical concentrations for interpreting pDST results when this survey was launched.^20^ Following the publication of the pretomanid critical concentrations by WHO, we enquired whether HPCs, where pretomanid was approved or used programmatically, were now planning to start validating pDST for this drug, if the drug was not already being tested or being validated.

The respondents were asked to report the availability of any commercial gDST assay, irrespective of whether it was a WHO-recommended molecular rapid TB diagnostic (mWRD) or had Conformité Européenne–In Vitro Diagnostic (CE-IVD) marking.^21^ Laboratory-developed tests/in-house assays, except WGS, were beyond the scope of this survey. All results were analysed in aggregate using three denominators. First, we used the number of countries, territories, and areas for all drugs. Second, we utilised the estimated TB incidence for 2024 for first-line drugs, including moxifloxacin, which is now recommended for the treatment of drug-susceptible TB.^1,22^ Even though levofloxacin is a second-line drug for treating RR-TB as well as rifampicin-susceptible, isoniazid-resistant TB, resistance to moxifloxacin can be inferred more accurately by testing levofloxacin.^1,18,23^ Therefore, we also selected the estimated TB incidence for 2024 for levofloxacin and fluoroquinolones as a class. Third, we used the estimated RR-TB incidence for 2024 for the remaining second-line drugs.^16^ No separate incidence estimates are published for Kosovo by the WHO. Pretomanid as part of the BPaL(M) regimen is not approved in eight countries in the WHO European Region (Albania, Armenia, Bosnia and Herzegovina, Israel, Montenegro, the Russian Federation, Serbia, and Türkiye) but is used programmatically in Armenia (Armenian National TB Programme, personal communication).^24^ The remaining seven countries were excluded when analysing the pDST and WGS results for pretomanid as routine DST is not currently needed in those settings for routine patient care. Pretomanid was considered relevant for Greenland for this analysis as Greenlandic health authorities can authorise the use of pretomanid if RR-TB is diagnosed.

### Ethical statement

Because this study included only anonymised survey data with no personal or sensitive data, ethical approval was not required.

## RESULTS

### pDST availability

Two EU/EEA countries and one other country, territory, or area (6% of the total countries, territories, or areas that account for 0.2% of the estimated TB incidence) did not carry out any pDST but referred the samples abroad for pDST (Figure 1A). The availability of pDST (Figure 1B) exceeded 97% of the incidence of TB or RR-TB for all countries, territories, or areas for 10 of the 16 drugs, including bedaquiline, clofazimine, delamanid, linezolid, and moxifloxacin. Levofloxacin pDST was available for 93% but, taking moxifloxacin into consideration, the availability for fluoroquinolones as a class was 99%.

**Figure 1.**
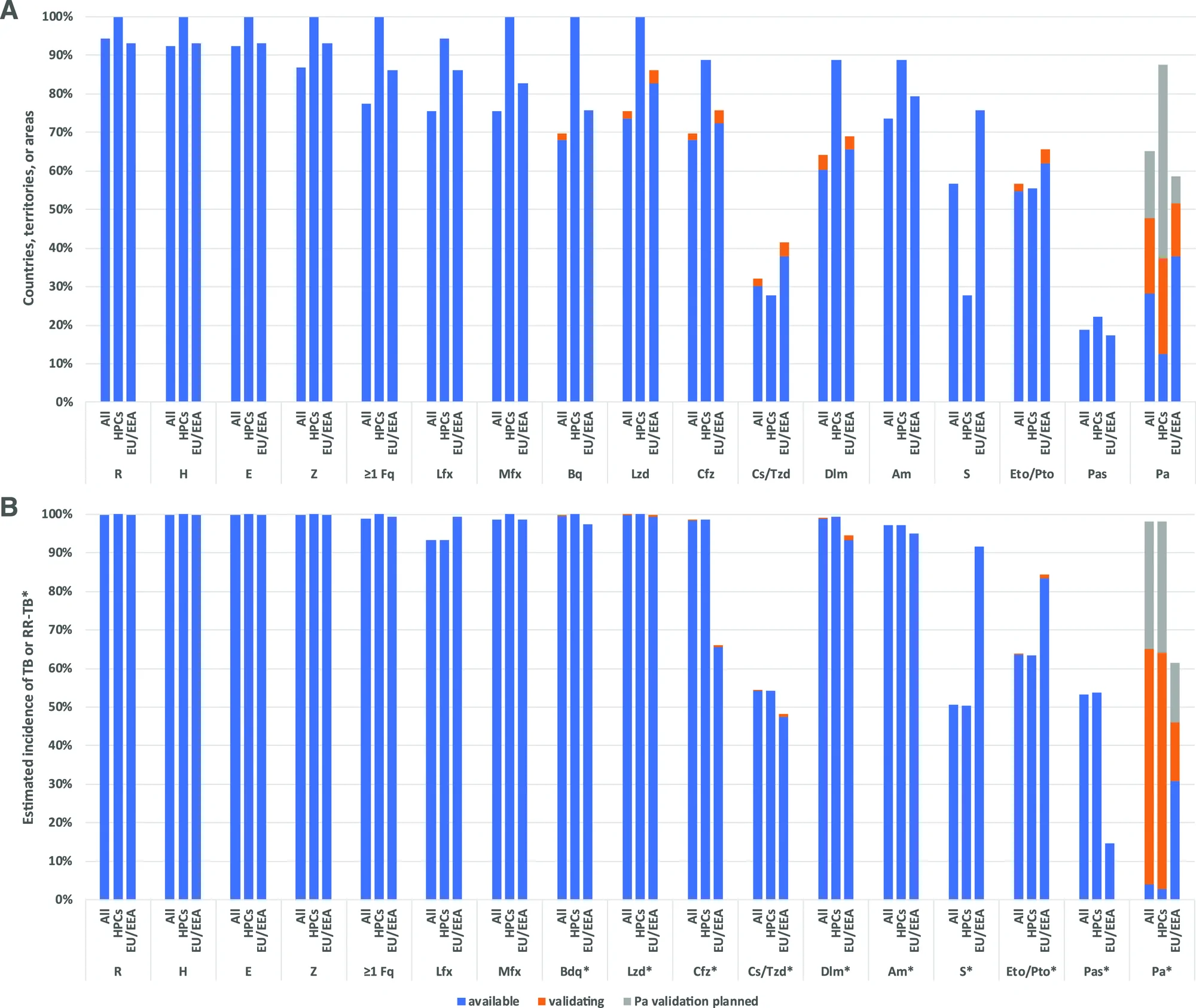
Availability of pDST for patient care. The estimated incidence of RR-TB was used for the drugs with an asterisk, whereas the estimated incidence of TB was used for the remaining ones. Am = amikacin; Bdq = bedaquiline; Cfz = clofazimine; Cyc = cycloserine; Dlm = delamanid; E = ethambutol; Eto = ethionamide; Fq = fluoroquinolone; H = isoniazid; Lfx = levofloxacin; Lzd = linezolid; Mfx = moxifloxacin; Pa = pretomanid; Pas = *para*-aminosalicylic acid; pDST = phenotypic drug susceptibility testing; Pto = prothionamide; R = rifampicin; RR-TB = rifampicin-resistant TB; S = streptomycin; Tzd = terizidone; Z = pyrazinamide.

The availability of pDST for cycloserine/terizidone, ethionamide/prothionamide, *para*-aminosalicylic acid, and streptomycin varied between 50% and 64% of the estimated RR-TB incidence, likely due to a combination of epidemiological, technical, and policy factors. Only 28% of HPCs tested streptomycin as the rates of resistance to this agent are high in eastern Europe for RR-TB.^25,26^ High rates of ethionamide/prothionamide resistance in eastern Europe coupled with the fact that pDST, as currently conducted, cannot reliably detect low-level resistance likely contributed to the lower availability of pDST for ethionamide/prothionamide, although other factors may have played a role.^18,25^ Finally, the critical concentration for cycloserine has only been recently published by the WHO and pDST for *para*-aminosalicylic acid is no longer recommended by the WHO.^21^

Pretomanid pDST was only available for 4% of the estimated RR-TB incidence in countries where pretomanid is approved or used programmatically (Figure 1B). Ongoing validation efforts of pretomanid pDST stand to benefit 61% of estimated RR-TB incident cases, and planned validations would cover a further 33% of RR-TB incidence, bringing the total to 98%, although this prediction will have to be verified in future surveys. Notably, even where pretomanid pDST was available, results were either not interpreted because no breakpoint existed for the method used (i.e., only the minimal inhibitory concentration was reported) or interpreted using different breakpoints depending on the country, territory, or area, although the latter factor likely resulted in few differences in practice, as RR-TB is rarely caused by *M. tuberculosis* lineage 1 in the WHO European Region.^20^ The recent publication of two WHO critical concentrations for pretomanid (i.e., 0.5 and 2 mg/L for the Mycobacterial Growth Indicator Tube [MGIT]) and the fact that the European Committee for Antimicrobial Susceptibility Testing and the European Medicine Agency now recommend a single screen value for pretomanid (i.e., 2 mg/L using MGIT) is expected to improve the standardisation of pDST and its interpretation.^20^

### Rapid gDST availability

An mWRD or other rapid gDST assay, excluding WGS, was available to detect resistance to rifampicin, isoniazid, and fluoroquinolones for more than 99% of estimated incidence in all countries, territories, or areas (Figure 2B). Two mWRDs by Cepheid (Xpert MTB/RIF and Xpert MTB/RIF Ultra) and two by Bruker/Hain Lifescience (GenoType MTBDR*plus* VER 2.0 and GenoType MTBDR*sl* VER 2.0) were available in between 75% and 91% of countries, territories, or areas (Supplementary Data Figure S1). The third mWRD by Cepheid (Xpert MTB/XDR) was used in 60% of countries, territories, or areas, whereas the remaining mWRDs, including the recently recommended tNGS assays (GenoScreen Deeplex Myc-TB and Oxford Nanopore Technologies AmPORE TB) were available in less than 24% of countries, territories, or areas and were being validated in less than 14% of countries, territories, or areas.

**Figure 2.**
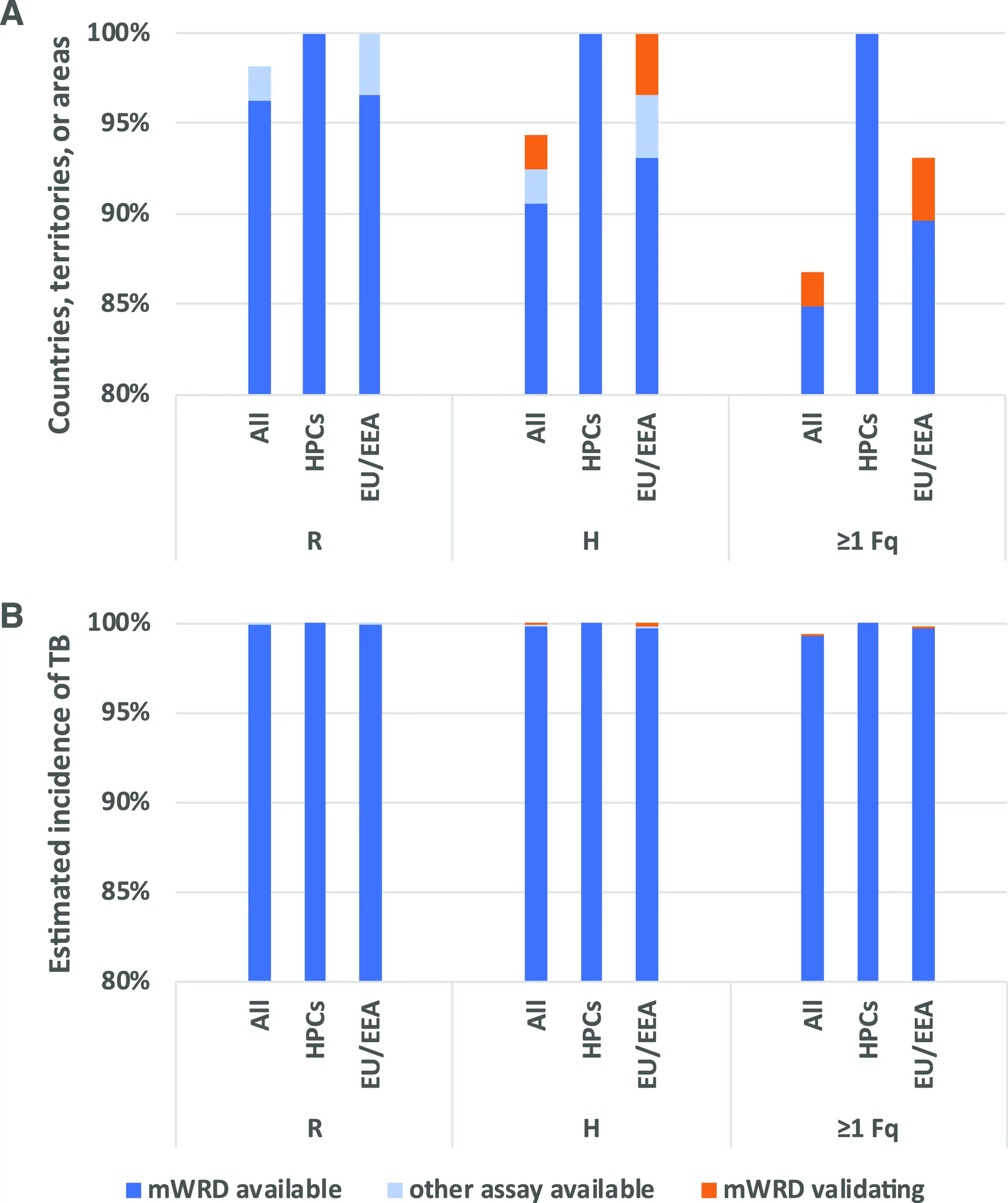
Availability of mWRD and other rapid gDST for patient care. EU/EEA = European Union/European Economic Area; Fq = fluoroquinolone; gDST = genotypic drug susceptibility testing; H = isoniazid; HPCs = high-priority countries; mWRD = WHO-recommended molecular rapid TB diagnostic; R = rifampicin.

### WGS availability

WGS requires more expertise and resources to be established and maintained than most other DST methods, which is reflected in the greater availability of this method in EU/EEA countries than in HPCs (i.e., 59% vs. only 11%, respectively [Figure 3A]). Only five of the 21 countries, territories, or areas where WGS was available harnessed its full potential by interpreting all drugs included in the survey. For some drugs, this might be partially linked to limitations of the WHO mutation catalogues (i.e., both editions do not cover cycloserine/terizidone and *para*-aminosalicylic acid, and the first edition does not include any resistance mutations for bedaquiline, clofazimine, and pretomanid^18^).

**Figure 3.**
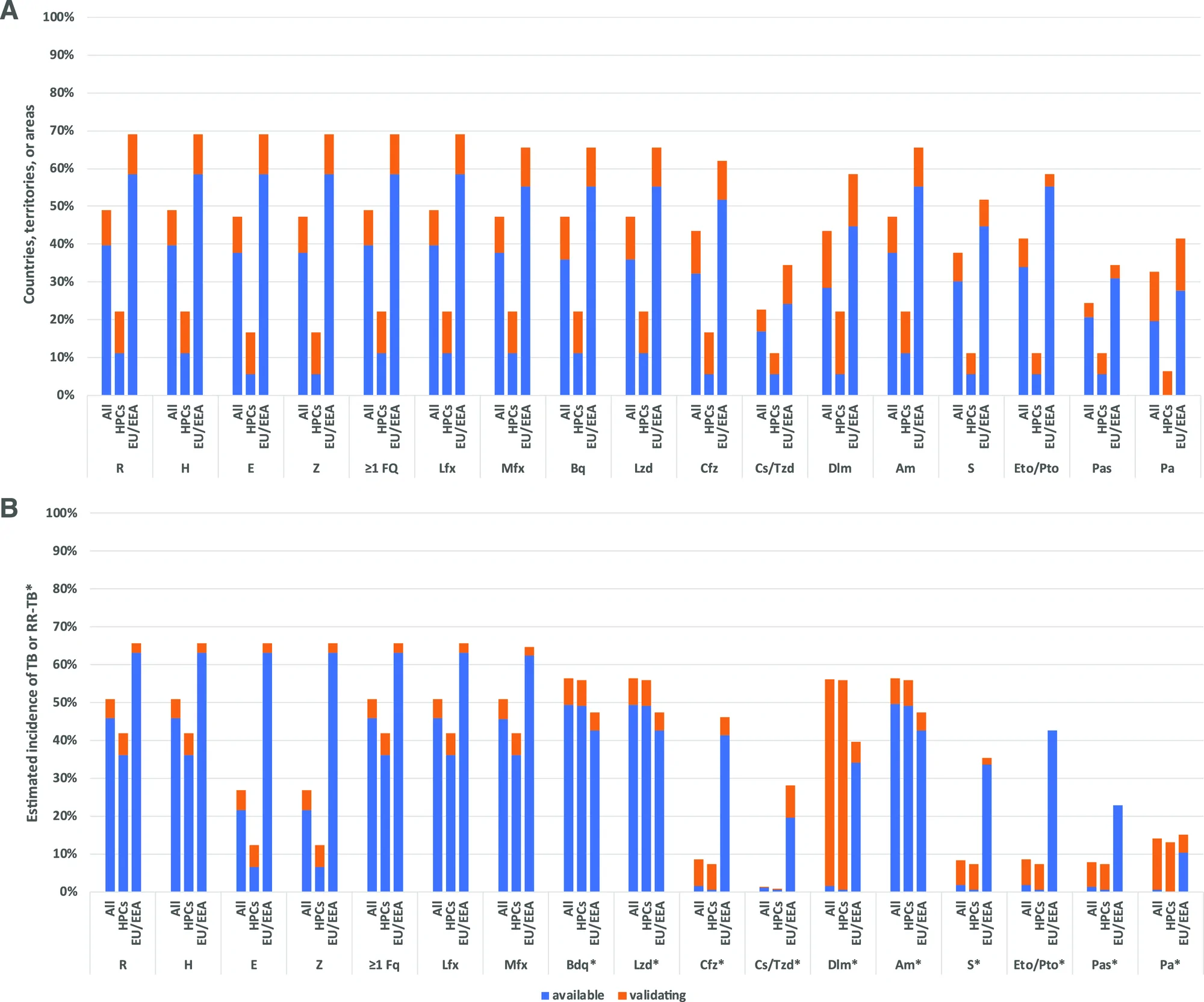
Availability of WGS for patient care. The estimated incidence of RR-TB was used for the drugs with an asterisk, whereas the estimated incidence of TB was used for the remaining ones. Am = amikacin; Bdq = bedaquiline; Cfz = clofazimine; Cyc = cycloserine; Dlm = delamanid; E = ethambutol; Eto = ethionamide; EU/EEA = European Union/European Economic Area; Fq = fluoroquinolone; H = isoniazid; HPCs = high-priority countries; Lfx = levofloxacin; Lzd = linezolid; Mfx = moxifloxacin; Pas = *para*-aminosalicylic acid; Pa = pretomanid; Pto = prothionamide; R = rifampicin; RR-TB = rifampicin-resistant TB; S = streptomycin; Tzd = terizidone; WGS = whole genome sequencing; Z = pyrazinamide.

## DISCUSSION

Only analysing the results using countries, territories, or areas as the denominator would provide a distorted picture of the DST availability because of the comparatively large number of small, low-incidence countries in western Europe. We minimised this bias by using the estimated incidence of all TB or RR-TB as the second denominator. We chose the estimated incidence figures rather than the number of notified persons to account for undiagnosed individuals but acknowledge that the accuracy of these figures varies depending on whether they rely on model, surveillance, and/or survey data.^16,22^ We analysed 53 countries, territories, and areas that responded to this survey and, to account for the uneven burden of TB within the WHO European Region, also focussed on two subsets: the 18 HPCs and 29 EU/EEA countries.

The optimal placement of a DST assay within a national (or equivalent) laboratory network depends on numerous factors, such as the national (or equivalent) diagnostic algorithm, local TB epidemiology, sample transportation capacities, and financial considerations. It may not be necessary for all TB reference laboratories, let alone regional laboratories, of every country, territory, or area with a low TB burden to carry out DST for all drugs.^14^ Indeed, establishing and maintaining quality-assured testing proficiency for a small number of samples can be challenging, and it may be more appropriate to refer relevant samples to specialised laboratories elsewhere. For example, three small countries, territories, or areas with a combined estimated TB incidence of fewer than 200 people, of which two countries had a rapid gDST assay available locally to detect multidrug-resistant TB, referred all samples abroad for pDST. Other countries, territories, or areas also availed themselves of specialised services elsewhere, although this was not systematically captured by this survey (e.g., the United Kingdom relied on pDST in Italy for bedaquiline and delamanid whilst validating pDST^27^). Nevertheless, we stress that national (or equivalent) and regional laboratories within a country, territory, or area have an important role to play in providing rapid gDST in HPCs, particularly if those are large, high-incidence countries.

To avoid misleading conclusions for pretomanid, it was important to exclude the seven countries in which BPaL(M) is not approved or used programmatically from the analysis, particularly as these countries (Albania, Bosnia and Herzegovina, Israel, Montenegro, the Russian Federation, Serbia, and Türkiye) account for 48% of the estimated RR-TB incidence of the WHO European Region. We note, however, that even where pretomanid is approved, it may not be in use, resulting in an overestimate of the current need for DST, which may apply to other second-line agents in some settings as well.^28^ These limitations notwithstanding, this survey revealed that 4 years after pretomanid was recommended by WHO, pDST was only available for 4% of the estimated RR-TB incidence for countries, territories, and areas where pretomanid is approved or used programmatically.^29^ Because the cross-resistance between delamanid and pretomanid appears to be high, existing delamanid pDST capacity may be considered to infer pretomanid resistance as an interim solution.^18^ However, pDST for pretomanid is still needed to detect exceptions to this general pattern. Therefore, greater financial, technical, and programmatic support is needed to reduce the time between the drug introduction and the availability of robust DST services. Furthermore, continued educational efforts are needed to ensure the overall quality of all laboratory procedures, including the correct result interpretation of novel diagnostics and discordant results that can impact the final test result.^30–32^

We would like to emphasise that this survey only covered the availability of DST and did not seek to quantify which proportion of relevant individuals are diagnosed with the different DST options, nor whether this occurs in a timely and quality-assured manner. In the United Kingdom, for instance, WGS is used for all new mycobacterial cultures for identification, gDST, and the elucidation of transmission clusters.^33^ As a result, pDST is no longer carried out for the vast majority of *M. tuberculosis* complex cultures, whereas rapid gDST directly from primary samples is available but only carried out selectively upon request. In HPCs, by contrast, gDST is widely used as the primary diagnostic method, whereas WGS, if available at all, is mostly used for RR-TB, although for selected cases only and often intermittently because of challenges linked to sustainable financing, instrument maintenance, and other factors.

Our survey has several additional limitations. First, we did not examine where DST assays were available within each country, territory, or area (i.e., whether at the reference laboratory and/or elsewhere). Second, the availability of some rapid gDST assays may have been missed in countries, territories, or areas where individual laboratories decide which assays to adopt (e.g., in Germany or Italy). Third, we did not capture the impact of reagent stockouts, and interruptions caused by maintenance issues. Fourth, we did not assess the quality of DST, which depends on the performance of individual laboratories and in some cases the assay manufacturer (e.g., in May 2025, BD expanded the recall of its MGIT PZA kit, the only pDST method approved for pyrazinamide^34^). Fifth, we did not address to what extent laboratories and clinicians are able to identify and overcome DST errors and correctly interpret discordant DST results.^31^ Finally, evaluating the timely and correct use of DST results to select and adapt regimens by clinicians was beyond the scope of this survey.^35^

## Supplementary Material

## References

[1] World Health Organization. WHO consolidated guidelines on tuberculosis: module 4: treatment and care. Geneva: WHO, 2025. 40163610

[2] Gupta A, Global adoption of 6-month drug-resistant TB regimens: projected uptake by 2026. PLoS One. 2024;19(1):e0296448. 10.1371/journal.pone.0296448 PMC10769048 38180980

[3] Köser CU, Maurer FP, Kranzer K. ‘Those who cannot remember the past are condemned to repeat it’: drug-susceptibility testing for bedaquiline and delamanid. Int J Infect Dis. 2019;80:S32-S35. 10.1016/j.ijid.2019.02.027 30818049

[4] Bateson A, Ancient and recent differences in the intrinsic susceptibility of *Mycobacterium tuberculosis* complex to pretomanid. J Antimicrob Chemother. 2022;77(6):1685-1693. 10.1093/jac/dkac070 PMC9155602 35260883

[5] Timm J, Baseline and acquired resistance to bedaquiline, linezolid and pretomanid, and impact on treatment outcomes in four tuberculosis clinical trials containing pretomanid. PLoS Glob Public Heal. 2023;3(10):e0002283. 10.1371/journal.pgph.0002283 PMC10584172 37851685

[6] Vambe D, Targeted next-generation sequencing implementation in Eswatini identifies rifampicin and bedaquiline resistance undetected by routine diagnostic testing. Nat Commun. 2026;17(1):8227. 10.1038/s41467-026-73551-w PMC13462916 42270607

[7] Ardizzoni E, Phenotypic and genotypic resistance to bedaquiline in patients with multi-drug-resistant tuberculosis-experiences from Armenia. Antimicrob Agents Chemother. 2025;69(5):e01839-24. 10.1128/aac.01839-24 PMC12057352 40202335

[8] Mallick JS, Acquired bedaquiline resistance during the treatment of drug-resistant tuberculosis: a systematic review. JAC-Antimicrob Resist. 2022;4(2):dlac029. 10.1093/jacamr/dlac029 PMC8963286 35356403

[9] Decroo T, Mesic A, Decuyper I. Longevity of modified standard short treatment regimens for rifampicin-resistant tuberculosis. Lancet Infect Dis. 2024;24(10):1069-1071. 10.1016/S1473-3099(24)00294-9 38880113

[10] Perumal R, Baseline and treatment-emergent bedaquiline resistance in drug-resistant tuberculosis: a systematic review and meta-analysis. Eur Respir J. 2023;62(6):2300639. 10.1183/13993003.00639-2023 PMC11035900 37945030

[11] Farooq HZ, Limited capability for testing *Mycobacterium tuberculosis* for susceptibility to new drugs. Emerg Infect Dis. 2021;27(3):985-987. 10.3201/eid2703.204418 PMC7920658 33622487

[12] Maurer FP, Diagnostic capacities for multidrug-resistant tuberculosis in the World Health Organization European region: action is needed by all member states. J Mol Diagn. 2022;24(11):1189-1194. 10.1016/j.jmoldx.2022.07.005 PMC12179496 35964846

[13] Günther G, Availability and costs of medicines for the treatment of tuberculosis in Europe. Clin Microbiol Infec. 2023;29(1):77-84. 10.1016/j.cmi.2022.07.026 PMC9801521 35961488

[14] Friesen I, Re: ‘availability of drugs and resistance testing for BPaLM regimen for rifampicin-resistant tuberculosis in Europe’ by Lange et al. Clin Microbiol Infect. 2024;30(9):1204-1206. 10.1016/j.cmi.2024.06.001 38851424

[15] Kherabi Y, Treatment outcomes of extensively drug-resistant tuberculosis in Europe: a retrospective cohort study. Lancet Reg Health Eur. 2025;56:101380. 10.1016/j.lanepe.2025.101380 PMC12281379 40697337

[16] World Health Organization. WHO MDR/RR-TB burden estimates. https://extranet.who.int/tme/generateCSV.asp?ds=mdr_rr_estimates (Accessed 15 January 2026).

[17] Yusuf E, Schijffelen MJ, Leeflang M. How to verify and validate a clinical microbiology test before it can be used in routine diagnostics: a practical guide. Clin Microbiol Infect. 2024;30(10):1261-1269. 10.1016/j.cmi.2024.06.028 38977077

[18] World Health Organization. Catalogue of mutations in *Mycobacterium tuberculosis* complex and their association with drug resistance. 2nd ed. Geneva: WHO, 2023.

[19] Lundeberg EE, *In vitro* activity of new combinations of β-lactam and β-lactamase inhibitors against the *Mycobacterium tuberculosis* complex. Microbiol Spectr. 2023;11(5):e01781-23. 10.1128/spectrum.01781-23 PMC10580993 37737628

[20] Köser CU. More nuance and transparency are needed when setting breakpoints for antimicrobial susceptibility testing of pretomanid. Clin Microbiol Infect. 2025;31(5):692-695. 10.1016/j.cmi.2024.12.035 39746445

[21] World Health Organization. WHO operational handbook on tuberculosis: module 3: diagnosis. Geneva: WHO, 2025.

[22] World Health Organization. WHO TB burden estimates. https://extranet.who.int/tme/generateCSV.asp?ds=estimates (Accessed 15 January 2026).

[23] World Health Organization. Optimized broth microdilution plate methodology for drug susceptibility testing of *Mycobacterium tuberculosis* complex. Geneva: WHO, 2022.

[24] TB Alliance. Access to the BPaL regimen. https://www.tballiance.org/access-countries/ (Accessed 7 February 2025).

[25] Merker M, Transcontinental spread and evolution of *Mycobacterium tuberculosis* W148 European/Russian clade toward extensively drug resistant tuberculosis. Nat Commun. 2022;13(1):5105. 10.1038/s41467-022-32455-1 PMC9426364 36042200

[26] Wollenberg KR, Whole-genome sequencing of *Mycobacterium tuberculosis* provides insight into the evolution and genetic composition of drug-resistant tuberculosis in Belarus. J Clin Microbiol. 2017;55(2):457-469. 10.1128/JCM.02116-16 PMC5277515 27903602

[27] UK Health Security Agency. Guidance national mycobacterium reference service-south: user handbook (updated 25 August 2023). https://www.gov.uk/government/publications/national-mycobacterium-reference-laboratory-nmrl-user-manual/nmrs-south-user-handbook (Accessed 3 October 2024).

[28] Otto-Knapp R, Availability of drugs for the treatment of multidrug-resistant/rifampicin-resistant tuberculosis in the World Health Organization European region, October 2023. Eurosurveillance. 2024;29(17):2400211. 10.2807/1560-7917.ES.2024.29.17.2400211 PMC11063671 38666403

[29] World Health Organization. WHO consolidated guidelines on tuberculosis: module 4: treatment: drug-resistant tuberculosis treatment. Geneva: WHO, 2020. 32603040

[30] Homolka S, Introduction of quality management in a National Reference Laboratory in Germany. PLoS One. 2019;14(10):e0222925. 10.1371/journal.pone.0222925 PMC6793863 31613905

[31] Köser CU, Guidance is needed to mitigate the consequences of analytic errors during antimicrobial susceptibility testing for TB. Int J Tuberc Lung Dis. 2021;25(10):791-794. 10.5588/ijtld.21.0428 34615575

[32] Anthony RM, Experiences from 4 years of organization of an external quality assessment for *Mycobacterium tuberculosis* whole-genome sequencing in the European Union/European Economic Area. Microbiol Spectr. 2023;11(1):e02244-22. 10.1128/spectrum.02244-22 PMC9927412 36475728

[33] Samuelsen Ø, The role of whole genome sequencing in antimicrobial susceptibility prediction of bacteria: 2025 update from the EUCAST subcommittee. Clin Microbiol Infect. 2026;32(7S):S1-S34. 10.1016/j.cmi.2026.05.012 42142806

[34] BD. Urgent: medical device correction - expansion. Type of action: product removal – discarded by customer BD BACTECTM MGITTM 960 PZA Kit. https://bdx.my.salesforce.com/sfc/p/#30000000Y0Yr/a/Ho000001rSpM/yL62cXsX2KyAfMfCa5GywLK9AA6qzWNmfe7U2eOUks0 (Accessed 30 May 2025).

[35] Larsson L, Further effort is needed to avoid irrational use after drug susceptibility testing for drug-resistant TB. Int J Tuberc Lung Dis. 2025;29(1):35-37. 10.5588/ijtld.24.0263 39754277

