# Supplementary material for: Availability of genotypic and phenotypic drug susceptibility testing for Mycobacterium tuberculosis in WHO European Region
Source: IJTLD Open. 2026 Oct 12;3(10):662–9. doi: 10.5588/ijtldopen.25.0600 (PMC13657020; doi:10.5588/ijtldopen.25.0600)
Supplement: Supplementary file 1 [file ijtldopen25-0600_supplementarydata1.pdf]

Data Figure S1 Availability of rapid gDST

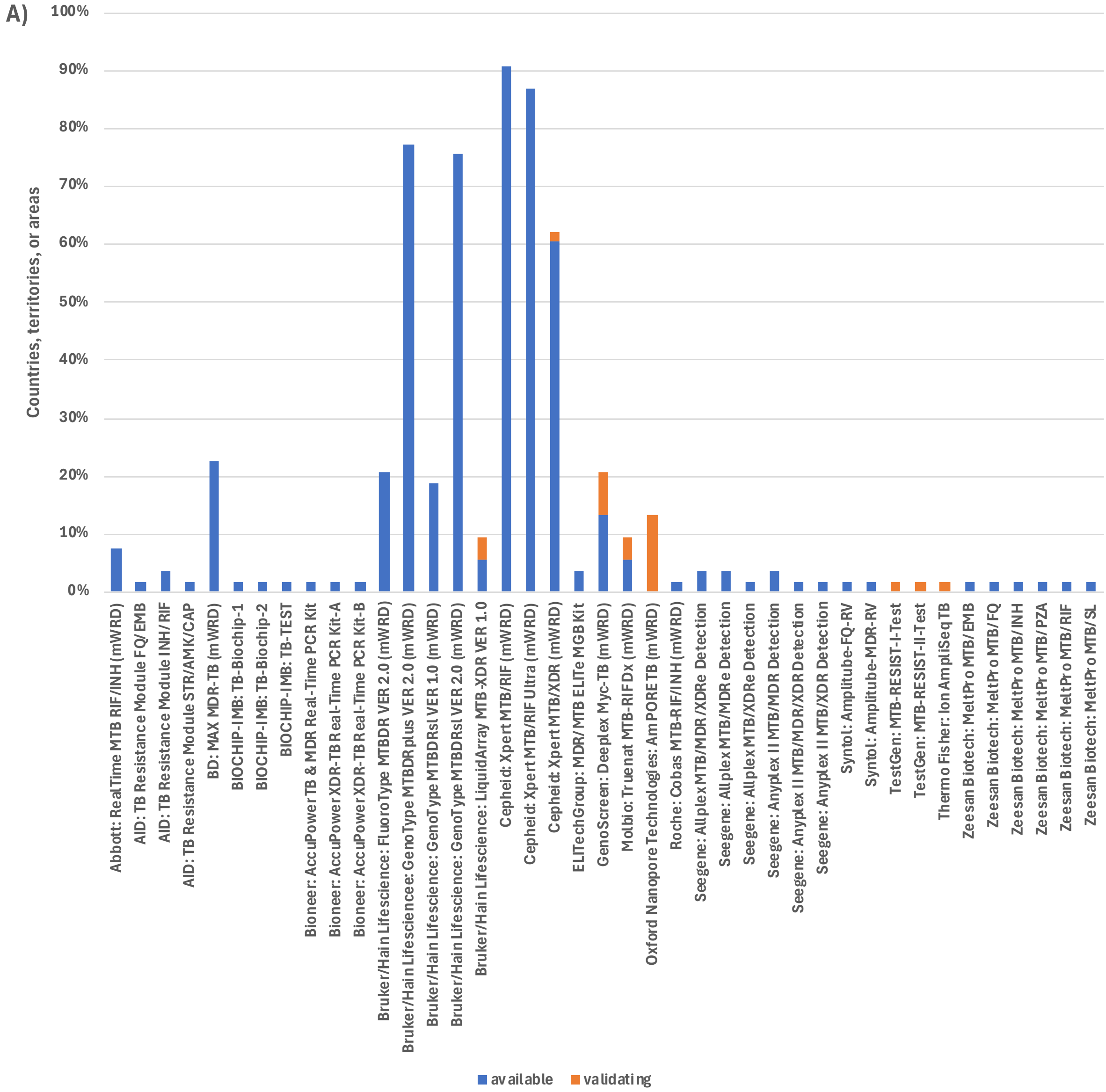

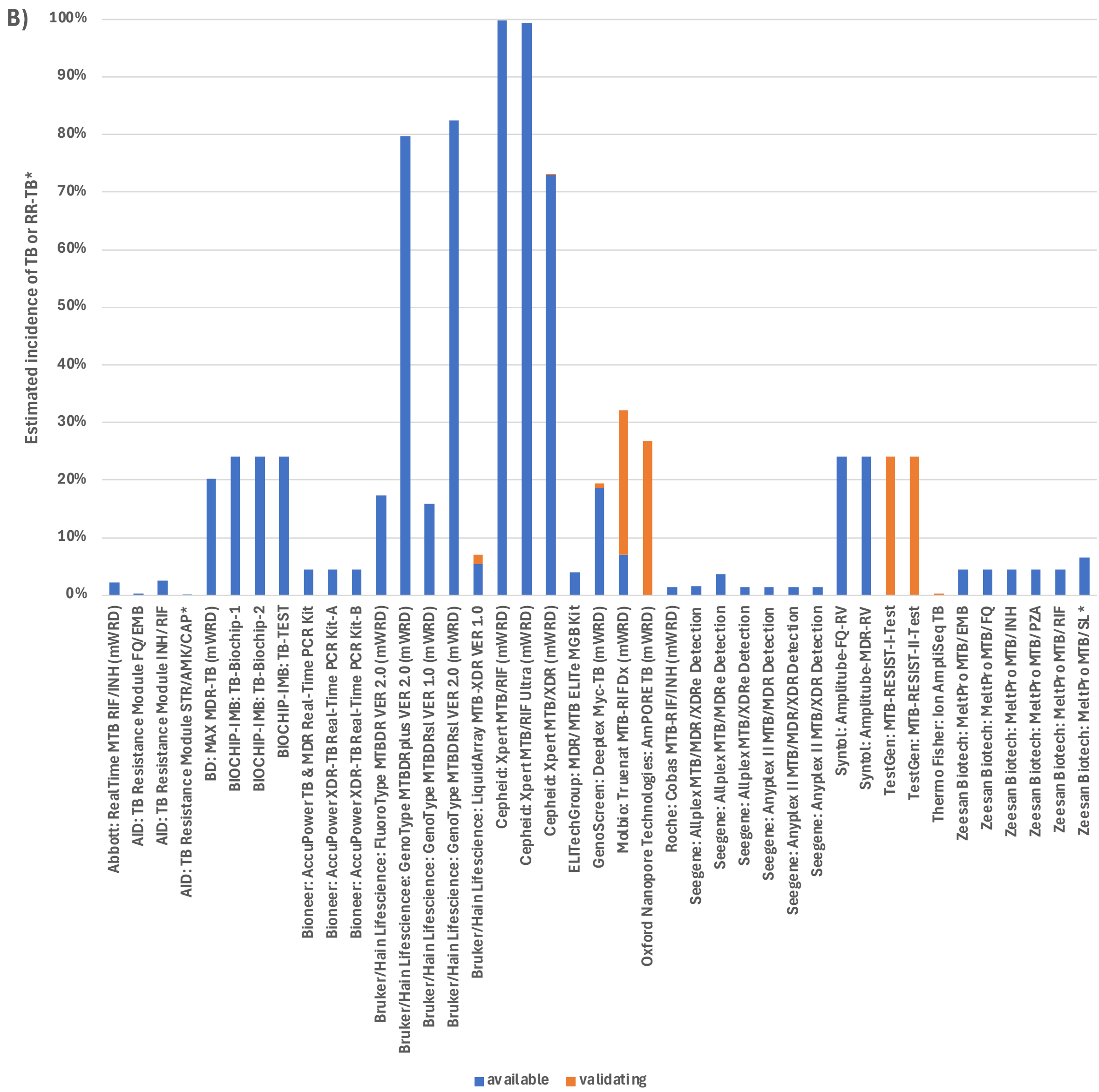

mWRD: WHO-recommended molecular rapid TB diagnostic. The estimate incidence of RR-TB was used for the assays with an asterisk, whereas the estimated incidence was used for the remaining ones.
